# Assessing Sexual Sadism in Sexual Homicide Offenders Using Behavioural Dimensional Scales, Typological Approaches, and Categorical Diagnoses

**DOI:** 10.5964/sotrap.14281

**Published:** 2025-10-21

**Authors:** Lara Consigliere, Lilith Zoe Maximiliane Kneißler, Aleshia Nanev, Michael Davis, Rajan Darjee

**Affiliations:** 1 Department of Clinical Psychological Science, Maastricht University, Maastricht, The Netherlands; 2 Centre for Forensic Behavioural Science (CFBS), Swinburne University of Technology, Melbourne, Australia; 3 Forensic Behavioural Assessment and Consultation Services (FBACS), Melbourne, Australia; Saint Mary's University, Halifax, NS, Canada

**Keywords:** sexual homicide, sexual sadism, paraphilias, assessment, Australia, New Zealand

## Abstract

Sexual sadism has been central to conceptualizing sexual homicide typologically, diagnostically, and behaviourally. Although about a third of cases involve sexual sadism, its reliable ascertainment is challenging, which is concerning given consequences of making or missing the diagnosis. This study examined 297 sexual homicide cases from Australia and New Zealand recruited from official online legal sources and evaluated the application of seven sexual sadism measures (clinical and research diagnoses, two typological approaches, and three behavioural scales). There were high correlations between all measures except clinical diagnosis. We confirmed scale and inter-rater reliability, convergent and concurrent validity, and external validity of behavioural scales. Rates of sexual sadism ranged from 4% for clinical diagnosis to 45% using the recommended Sexual Sadism Scale (SeSaS) Part 1 cut-off of 4. But other approaches identified between 1 in 5 and 1 in 3 cases. Cut-off scores of 5 on the SeSaS Part 1, 6 on the Sexual Homicide Crime Scene Rating Scale for Sexual Sadism (SADSEX-SH), and 6 on the Massachusetts Treatment Centre Sadism Scale (MTCSS) appeared equivalent and identified around 30% of cases. Offence and offender characteristics correlated with sadism ascertained with the SeSaS and SADSEX-SH were equivalent and as expected from the literature. We conclude that the SeSaS and SADSEX-SH are equivalent scales, but the recommended SeSaS Part 1 cut-off of 4 may be too low in sexual homicide cases. We make practical recommendations for a structured professional judgment approach to diagnosing sexual sadism disorder.

## Abstract (plain language summary)

**Background:**

Sexual homicide and sexual sadism have been widely researched. Research has found that sexual sadism is present in about 1 in 3 cases of sexual homicide. However, the role that sexual sadism plays has been difficult to pinpoint due to a number of issues relating to the definition and assessment of sexual sadism. In general, three different approaches to ascertain sexual sadism can be used: the most common one, especially in clinical practice, is a categorical clinical approach. Categorical approaches (e.g., the ICD-11) view sexual sadism as a disorder that is either present or absent. However, recent research has provided evidence for the dimensionality of sexual sadism, meaning that the disorder exists on a continuum where features can range from minor to severe. As a consequence, researchers have developed dimensional scales that look at behavioural markers to assess the presence and severity of sexual sadism. A different approach is to apply typologies, where an offender’s set of personal characteristics or behaviours are used to classify them into one of several groups, one of which is a sadistic group.

**Why was this study done?:**

This study was developed to ascertain the most reliable measure of sexual sadism in sexual homicide offenders, and to address the lack of clear guidelines on how to use these tools and approaches in practice.

**What did the researchers do and find?:**

The study compared three different approaches to sexual sadism: a categorical approach, a typological approach, and a dimensional approach. For the categorical approach, we looked for evidence of a diagnosis of sexual sadism disorder made by a clinician (clinical diagnosis), and we used the ICD-11 manual to assess the presence of coercive sexual sadism disorder (research diagnosis). For the typological approach, we identified offenders who could be grouped into two different sadistic groups (the sexualised murderer and sadistic rapist/child molester typologies). Finally, the dimensional approach consisted of the use of three behavioural scales (the SeSaS, SADSEX-SH, and MTCSS). So, we used seven different measures of sexual sadism for our analyses.

We ascertained whether these measures identified the same proportion of sexual sadists, the same population, and the same type of sexual sadism. We also tested how adopting different approaches to some of these measures (e.g., changing the cut-off of the scales or removing certain items) led to different results. We found that using a SeSaS Part 1 cut-off of 5, a SeSaS Part 1 cut-off of 4 after removing one item, a SeSaS Part 2 cut-off of 2, a SADSEX-SH cut-off of 6, a MTCSS cut-off of 6, and the sexualised murderer typology were the approaches that identified prevalence rates of sexual sadism that fell within the expected range (about 1 in 3 cases). The SeSaS and the SADSEX-SH were found to be very similar and to show good reliability and validity.

**What do these findings mean?:**

Our findings indicate that the SeSaS and the SADSEX-SH can be considered equivalent scales that can be used interchangeably. Considering that the SeSaS has been the most researched behavioural scale so far and that it is the only one that – to our knowledge – is used in practice, when diagnosing sexual sadism among sexual homicide offenders we recommend using the SeSaS with a cut-off of 5 in Part 1 alongside a typological approach. The clinician or researcher assessing the presence of sexual sadism should also incorporate their professional judgment in a structured way.

## Highlights

- The SeSaS Part 1 cut-off of 4 could be overinclusive in SH cases; a cut-off of 5 may be more appropriate.
- Cut-offs of 6 on the SADSEX-SH and MTCSS are also appropriate to use in SH cases.
- SeSaS Part 1 and SADSEX-SH appear to be equivalent measures of sexual sadism in SH cases.
- A structured professional judgment approach to diagnosing sexual sadism disorder should be followed in practice, using the SeSaS and a typological approach.

Sexual homicides are defined by the presence of sexual arousal or behaviour in association with the crime, either before, during or after killing, and they represent one of rarest forms of violent crimes (DeLisi et al., 2024). Sexual sadism has played a prominent role in conceptualising sexual homicide, and it has repeatedly been identified as a common and relevant clinical feature of sexual homicide offenders (SHOs; Darjee, 2019; DeLisi et al., 2024; Healey et al., 2013; Koch et al., 2011). Therefore, much literature on sexual homicides has focused on trying to understand the relationship between these crimes and sexual sadism.

## Sexual Sadism: A Clinical Diagnosis

The Diagnostic and Statistical Manual of Mental Disorders, Fifth Edition (DSM-5; American Psychiatric Association [APA], 2013) and the International Classification of Diseases, 11^th^ Revision (ICD-11; World Health Organization, 2018) offer similar definitions of sadism, with DSM-5 using the term “sexual sadism disorder” (SSD), whilst ICD-11 uses “coercive sexual sadism disorder” (CSSD). Both describe a longstanding tendency to experience sexual pleasure from the infliction or observation of physical or psychological suffering in others. However, a problem of lack of reliability of categorical diagnoses is evident across studies (Marshall & Kennedy, 2003; Marshall et al., 2002).

Using DSM-5 or ICD-11, a diagnosis of sexual sadism can only be made if, among other criteria, there is evidence that sadistic fantasies, urges, or behaviours are a source of arousal (APA, 2013; World Health Organization, 2018). Self-reports are the main, if not only, source of information about sexual fantasies and preferences. But those who commit sexual violence may not disclose accurate information about their impulses, actions, and motivations (Beauregard et al., 2007). This can compromise reliability and validity of clinical diagnoses. In addition, clinicians may base diagnoses on intuition, inferences, or idiosyncratic criteria, not apply criteria properly or objectively, and not use sufficient information from external sources (Reale et al., 2017).

A further complicating factor is whether sexual sadism is naturally categorical or dimensional. Recent research provides strong evidence that sexual sadism is dimensional and at the high end of a longer dimension of coercive and aggressive sexual interests and behaviour, which has been called the “agonistic continuum” (Knight et al., 2013; Longpré, Guay, et al., 2018; Longpré, Proulx, et al., 2018; Longpré et al., 2020). This further explains problems with reliability, as any diagnostic threshold is arbitrary, with sexual sadism having different levels of severity and blurring into lesser degrees of sexual interest in coercion and aggression.

Therefore, to improve reliability and validity, it has been suggested that researchers and clinicians adopt a dimensional approach using objective behavioural data describing offences and crime scene actions to assess for the presence and degree of sexual sadism (Marshall & Hucker, 2006; Marshall & Kennedy, 2003).

### Dimensional Scales

In 2009, Nitschke and colleagues developed the first dimensional scale assessing sexual sadism, the Sexual Sadism Scale (SeSaS). The SeSaS has two parts: Part 1 is an 11-item dimensional scale based on crime scene behaviours with a score of 4 or more suggestive of sexual sadism (Table 1). Part 2 has three further items, termed ‘additional indicators’ (see Table 1), which can help a practitioner to form a clinical judgment as to whether sexual sadism is present. In practice the SeSaS has become the benchmark for dimensional scales.

Myers and colleagues (2019) developed the Sexual Homicide Crime Scene Rating Scale for Sexual Sadism (SADSEX-SH), an 8-item rating scale designed to assess the presence and severity of sexual sadism among SHOs, with a suggested cut-off for identifying sexual sadism of 6, but there have been few studies of the SADSEX-SH. Longpré et al. (2019) developed the Massachusetts Treatment Center Sadism Scale (MTCSS), a 15-item scale aiming to capture the severity, rather than presence, of sexual sadism among sex offenders, so not cut-off has been suggested, and the MTCSS has only been studied in the development sample.

The SeSaS, the SADSEX-SH, and the MTCSS have shown good psychometric properties in terms of internal consistency, interrater reliability, discriminating power, and convergent validity (Longpré et al., 2019; Myers et al., 2019; Nitschke et al., 2009; Reale et al., 2020; Stefanska et al., 2019), but the SeSaS has the most validation data and is the only scale used in practice.

### Limitations of the SeSaS

The SeSaS recommended cut-off score of 4 derives primarily from the initial sample (Nitschke et al., 2009). Although it has demonstrated good sensitivity and specificity in three samples (Nitschke et al., 2013), the rate of SeSaS sexual sadism identified in most samples of sexual offenders (including SHOs) is lower than the 50% set in the development sample. In addition, a maximum score of 11 has never been replicated by other researchers, with most finding highest scores ranging from 7 to 10 (e.g., Darjee, 2019; Longpré, Proulx, et al., 2018; Stefanska et al., 2019). It has also been suggested that the cut-off of 4 is too low (Gonçalves et al., 2020; Higgs et al., 2019; Mokros et al., 2012; Stefanska et al., 2019).

Secondly, Item 1 of the SeSaS, identifying sexual arousal during the crime, may be problematic. Although relevant in sexual sadism, sexual arousal is obviously common across sexual offences and is not specific to sexual sadism. Studies have identified rates of 50% (Nitschke et al., 2009) to 96.1% (Darjee, 2019; see Table 9 in the Electronic Supplementary Materials) in sexual homicide and sexual offending samples. Therefore, considering sexual arousal during the crime an indicator of sexual sadism might result in inflated scores on the scale, albeit that such arousal is necessary to identify any paraphilia.

Thirdly, certain items of the SeSaS, such as expressive violence and use of power and control, are very likely to be endorsed in sexual homicide cases, regardless of whether the offender is driven by sadistic sexual arousal. This may lead to higher baseline SeSaS scores in sexual homicide cases. Along with the ease with which Item 1 is endorsed, a score of three is very easily achieved in a sexual homicide case, only requiring one more item to achieve the recommended threshold score of 4.

Finally, Part 2 of the SeSaS has been under researched. Most research on the SeSaS has focused on Part 1 (see Electronic Supplementary Materials, Tables 9 and 10). Only two studies have looked at Part 2 (Clarkson et al., 2020; Mokros et al., 2014) and none have examined the use of the final structured judgment. In general, there is limited guidance for practitioners on how to integrate Part 1 and Part 2, and limited data on the prevalence of Part 2 items.

### Typological Approaches

Classification system approaches (i.e., typologies) can be used to disentangle, understand, and organise the heterogeneity of sexual offending (Robertiello & Terry, 2007). Over the last fifty years, several typologies of sexual offenders and SHOs have emerged, and they all include a category which aligns with sexual sadism.

Following a systematic review, Higgs et al. (2017) classified three types of SHOs: sexualised, grievance, and rape murderers. One of the sexualised murderer’s distinguishing features was the presence of sadistic sexual fantasies, interests, and urges culminating in sexually sadistic behaviour. The MTC Rapist Typology Version 3 (MTC:R3; Knight et al., 1998) sadistic sexual type is characterised by sexual pleasure gained from aggressive acts, in this case the subjugation of the victim. Most typologies of sexual aggressors against women identify very similar types (Wojcik & Fisher, 2019) and there is an overlap between these types and those of SHOs (Oligny et al., 2023). The MTC Child Molester Typology Version 3 (MTC:CM3; Knight, 1989) recognises a group with a low level of contact with children, but who inflict a high degree of physical injury, who are high on sadism and other such typologies identify a sadistic type (Lanning, 2001).

Although not all cases fit neatly into the types defined in any typology, and intermediate types are seen, typologies can be useful in investigative and forensic assessment contexts, where they help practitioners organise the factors in a case, recognise patterns, and generate hypotheses. However, there is limited empirical evidence to attest to their validity, and their use in forensic practice is uncommon.

## The Link Between Sexual Sadism and Sexual Homicide

Prevalence rates of sexual sadism among SHOs vary considerably, with most findings ranging from 25% to 37% (Darjee, 2019; Hill et al., 2006; Reale et al., 2020; Stefanska et al., 2019). The broad range of rates is likely due to different definitions and approaches used to identify sexual sadism (Marshall & Kennedy, 2003; Marshall et al., 2002), in addition to variations in sample characteristics.

Darjee (2019), Hill and colleagues (2006), and Stefanska and colleagues (2019) found sexual sadism appeared to be a particularly influential factor for sexual violence, sexual homicide, and serial sexual homicide. Three models of sexual homicide point towards a motivational and reinforcing relationship between sexual sadism and sexual homicide (Arrigo & Purcell, 2001; Hickey, 2002; Ressler et al., 1988), although these models are primarily based on serial sexual homicide.

It has also been suggested that sadistic SHOs are more likely to reoffend than non-sadistic SHOs (Hickey, 2002; Myers et al., 2023), and although prospective and pseudoprospective studies have not found sexual sadism specifically predicts recidivism (Eher et al., 2016), serial SHOs have higher rates of sexual sadism than single SHOs (James & Proulx, 2014; Reale et al., 2020; Stefanska et al., 2019). Therefore, a thorough understanding of sexual sadism and its role in sexual homicides will have important implications for the practical management of these cases.

An obstacle to this goal remains the lack of clear guidelines on how to define and assess sexual sadism. Although dimensional scales are now considered the best approach, only the SeSaS has extensive empirical evidence to back it and is used in some forensic practice settings internationally. There remain questions as to how to apply the SeSaS and the other scales in practice, what cut-off scores to use when assessing SHOs, how to integrate them with other approaches and indicators of sexual sadism, and how ultimately to make a diagnosis of SSD or CSSD.

Although sexual sadism, and the broader agonistic continuum, is dimensional, in practice categorical diagnoses will continue to be required until there is a universal shift towards a dimensional approach, as is occurring with personality disorders (Hopwood et al., 2018). Even then, thresholds will be required to define levels of severity (Darjee & Davis, 2025).

## The Present Study

The aim of the current study was to investigate how sexual sadism can be assessed and expresses itself in a large representative sample of completed (i.e., not attempted), prosecuted, and solved sexual homicide cases from Australia and New Zealand. We applied seven different measures of sexual sadism. Two were categorical: a diagnosis of SSD previously made by a clinician (not specifically for this study) and a diagnosis of ICD-11 CSSD made by the authors of this study. Two were typological: the sexualised murderer type (Higgs et al., 2017), and, depending on the victim’s age, the MTC:R3 sadistic sexual rapist (Knight et al., 1998) or MTC:CM3 sadistic child molester type (Knight, 1989). Finally, three were dimensional behavioural scales: the SeSaS (Nitschke et al., 2009); the SADSEX-SH (Myers et al., 2019); and the MTCSS (Longpré et al., 2019). The primary focus was on the dimensional scales, given research recommendations that they should be used in the assessment of sexual sadism. We aimed to do the following:

- For each dimensional scale, ascertain descriptive statistics, including the number of cases identified with different cut-off scores, internal consistency, inter-rater reliability, concurrent and convergent validity, and, for the SeSaS Part 1 and SADSEX-SH, external validity by examining associations with specific offender and offence characteristics.
- Ascertain the rates of sexual sadism identified by different diagnostic, typological, and dimensional behavioural approaches.
- Examine concurrent and convergent validity between the seven different measures, and for the three dimensional behavioural scales, ascertain equivalent cut-off scores.
- Ascertain correspondence between measures, i.e. whether they identify the same cases.
- Given the similarity and overlap between the SADSEX-SH and SeSaS Part 1, determine whether they are equivalent instruments.

Our primary goal was to help guide practitioners in the assessment of sexual sadism in SHOs, including which measures to use, what cut-off scores to adopt when applying dimensional scales, and how to integrate behavioural dimensional, typological, and diagnostic approaches. Secondarily, the study examined whether the presentation of sexual sadism in SHOs in Australia and New Zealand is similar to that found in previous research.

## Method

### Sample

The sample comprised 297 sexual homicide cases that occurred from 1918 to 2019 in Australian and New Zealand territory. The cases were obtained from publicly available websites containing official Court reports of legal cases, primarily the Australasian Legal Information Institute (AustLII) and New Zealand Legal Information Institute (NZLII), along with the searchable online databases of specific jurisdictions (Queensland, Tasmania, Western Australia, Northern Territory, and New Zealand). For each case, one or more Court reports were available including a description of the circumstances of the offence, victim and perpetrator characteristics (e.g., the presence of any medical diagnoses), the perpetrator’s arrest and depositions, applicable laws, and sentence.

To identify potential sexual homicide cases, terms relating to homicide (i.e., “homicide”, “murder”, or “manslaughter”) were combined with terms relating to the sexual aspect of the offence (i.e., “sex*”, “rape”, “semen”, “penis”, “breast*”, “genital*”, “vagina*”, “anal”, “anus”, “sodom*” “sadis*”, “naked”, “nude”, “molest*”, “indecen*”). Cases were then included if the offence met at least one of the Federal Bureau of Investigation (FBI) criteria for sexual homicide (Ressler et al., 1988): 1) the victim was found either partially or completely naked; 2) the genitals were exposed when the body was found; 3) the body was displayed in a sexually explicit position; 4) one or more objects had been inserted in bodily cavities; 5) there is evidence of sexual contact; 6) there is evidence of either substitute sexual activity or sadistic sexual fantasies. Where only the first criterion was met and there was no evidence of sexual activity having occurred, cases were not considered sexual homicides and excluded.

### Coding Procedure

Each case was coded using a coding sheet previously created and used by one of the senior researchers supervising the project (RD). The coding sheet included variables relating to the offence, offender, and victim based on Beauregard and Martineau (2013), Skott et al. (2019), Skott, Beauregard, and Darjee (2021), and Skott, Beauregard, et al. (2021), as well as additional variables based on recent literature findings on sexual homicide and the clinical and investigative experience of the senior researchers (RD and MD). Variables of interest used for the present study are presented below.

Cases were coded by masters- or doctoral-level forensic psychology or forensic psychiatry post-graduate students. All were trained by the two senior research supervisors (RD and MD) in the coding of the dataset and specifically in the application of measures and scales. Each researcher practiced and discussed with the supervisors the coding of six cases, for which the coding was then discarded. Weekly meetings were arranged to discuss or clarify any issues arising with one of the senior researchers (RD) and the other coders (cases were anonymised for this). Two researchers coded each case independently, so that inter-rater reliability could be determined. Intraclass correlation coefficients (ICC_A, 1_; two-way, random effects model, single ratings, absolute agreement method; McGraw & Wong, 1996; Shrout & Fleiss, 1979) were calculated for continuous variables and Kappa coefficients for categorical variables. The size of ICCs and Kappas were interpreted using, respectively, Shrout’s (1998) adaptation of the guidelines of Cohen (1960) and Landis and Koch (1977).

Each case was coded primarily from the official Court reports. Where variables could not be coded from legal documents, other sources of information about cases were accessed. These included news articles, crime websites, books, podcasts, videos, and academic papers. This is an approach which has been adopted in other research on unusual and rare crimes (Canter et al., 2004; Ferguson & Pooley, 2019; Gerard et al., 2007; Oostland & Brecht, 2020; Petreca et al., 2020; Porter & Alison, 2019, 2001; Quinet, 2011; Rosman & Resnick, 1989). Non-legal sources were only used if the details in them regarding variables available from legal sources were accurate. If a variable could not be coded from a legal or non-legal source, it was coded as missing. For every variable there was an associated coding of whether it had been ascertained from legal or non-legal sources or was missing. For each case there was also an overall rating of the quality of information available.

In cases where the victim was killed by more than one offender, variables relating to the offender were coded separately for each offender. In cases where the same offender killed multiple victims, victim variables were coded separately for each victim, while each offender variable was coded as present if its presence could be established for at least one of the homicides. The unit of analysis in the current study was the offender.

#### Measures of Sexual Sadism

##### Clinical and Research Diagnoses

If a clinician’s official diagnosis of SSD was documented in the Court report, then the offender was coded as having a clinical diagnosis of sexual sadism disorder (CDSSD). To ascertain the reliability of these diagnoses, the coding of this particular variable was exclusively based on the information available in the Court reports for that case (i.e., no secondary source of information was consulted). So, it had to be unambiguously reported that a psychiatrist or psychologist had diagnosed sexual sadism at some point in the offender’s life.

Each paraphilic disorder was coded following the ICD-11 manual (World Health Organization, 2018), namely by looking at whether the offender met the diagnostic criteria for the disorder, displayed behaviour consistent with the paraphilia at any of their offences, displayed behaviour consistent with the paraphilia outside of the offences as reported by them or others, there was evidence of fantasies consistent with the paraphilia, or there was evidence of a clinical or forensic diagnosis of the paraphilia. The scoring of the ICD-11 paraphilic disorders reflected the strength of evidence in the documents examined through four-level coding (i.e., no, possible, probable, or definite evidence). For a research diagnosis of coercive sexual sadism disorder (RDCSSD), there had to be probable or definite evidence that the offender met the diagnostic criteria for the disorder, although we also looked at how many cases were ascertained using the lower ‘possible’ threshold.

##### Dimensional Behavioural Scales

Each dimensional scale was coded following the developer’s guidelines. Where an item could not be coded due to missing information, the coding was changed to absent (0). SeSaS items were coded based on the sexual homicide(s) and other index and past offences. Each Part 1 and Part 2 item (see Table 1) was coded as either absent (0) or present (1; Nitschke et al., 2012). Only sexual homicide offences, index or past, were used to code the SADSEX-SH. Each of the eight SADSEX-SH items (see Table 1) was rated not present/unknown (0), possibly present/some evidence (1), or present (2; Myers et al., 2019). All the offender’s sexual crimes, including past and current homicides and non-fatal offences, were used to code the MTCSS. For the MTCSS, each of the 15 items (see Table 1) was coded absent (0) or present (1; Longpré et al., 2019).

##### Typologies

The Higgs et al. (2017) sexual homicide typology was coded using the authors’ description of the three types. So, each case was coded as ‘sexualised murderer’ if there was evidence that the “killing is functionally related to the sexual element of the offence” (coders were instructed not to code this type based on the scores obtained from the sexual sadism scales), ‘grievance murderer’ if the killing was “driven by angry schema and an excessively aggressive response style”, or ‘rape murderer’ if “only an indirect association between the sexual offence and killing” was found (Higgs et al., 2017, p. 10). This identified cases of interest who were considered to be sexualised murderer type (SMT).

The MTC:R3 (Knight et al., 1998) typologies were coded where victims were 14 years old or older. Each case was allocated to one of the five types: opportunistic, pervasively angry, vindictive, sadistic sexual or non-sadistic sexual. If the victim was under 14 years of age the MTC:CM3 (Knight, 1989) typology was coded across both aforementioned axes. Axis 2 “low contact” cases displaying a high level of physical injury (all of the homicide cases as the victims died) were coded as high or low in sadism. The former were considered in the ‘sadistic child molester’ type. A single variable, ‘MTC:R3/CM3 sadistic type’ (MTC:R3/CM3ST), was then created to capture whether a case was either a ‘sadistic sexual rapist’ or ‘sadistic child molester’ according to MTC:R3 or MTC:CM3. So, an offender identified as sadistic using either of these typologies, or rarely both if they had child and adult victims, was rated yes (1) for this variable. Typologies were rated before researchers rated the dimensional behavioural scales.

For typological and diagnostic ascertainment of sexual sadism cases with insufficient information for coding were assumed not to meet the requisite threshold for either having the diagnosis or being in a sadistic typological group.

#### Other Variables of Interest

##### Correlates of Sexual Sadism

A number of offender and offence variables were selected to identify the correlates of, and therefore externally validate, sexual sadism identified with the SeSaS Part 1 and the SADSEX-SH recommended cut-offs. These variables were coded dichotomously as present (1) or absent (0). Variables were selected based on previous research literature on correlates of sexual sadism (Darjee, 2019; Hill et al., 2006; Proulx et al., 2007; Reale et al., 2020). A few variables were added due to their relevance in other analyses (e.g., sample descriptives), or because we thought they might be linked to other correlates previously identified in the literature. These additional variables included paraphilic disorders, schizophrenia, and brain injuries; offender’s lifestyle (e.g., the use of substances); victim’s cause of death and whether they were targeted or stalked; involvement or use of weapons; long offences; whether the offender was a serial killer; and evidence of attempted suicide following the SH. Variables that were captured by items of the dimensional scales were not included in the bivariate analyses. All variables included in these analyses are listed in Tables 6 and 7 in the Electronic Supplementary Materials.

##### Personality and Paraphilic Disorders

The presence of psychopathic traits was assessed by applying the PCL:SV (Psychopathy Checklist: Screening Version; Hart et al., 1995) to all the information available about a case in legal and non-legal sources. ICD-11 criteria (World Health Organization, 2018) were applied to identify personality disorders (severity and traits) and paraphilic disorders, including coercive sexual sadism disorder (see above). To code the presence of a personality disorder, we used the general description of personality disorder provided by the manual and only selected the cases meeting the highest levels of moderate and severe personality disorder traits shown.

### Missing Data and Data Sources

Across all 172 variables and 297 cases, an average of 31.5 (18.2%) variables could not be coded per case, and an average of 9.8 (5.7%) variables were coded from non-legal sources only. So, the great majority of variables (94.3%) were coded from legal sources.

### Analysis

All statistical analyses were undertaken with IBM SPSS Statistics, version 28. Firstly, for the three dimensional scales we derived means, medians, standard deviations, and ranges. We used Cronbach’s alpha to determine internal consistency and ICCs for inter-rater reliability. We found that across the 172 variables used for our analysis, 138 (79.8%) showed substantial, almost perfect, or perfect inter-rater agreement, and only 2 (1.2%) showed slight or poor agreement. The agreement for the seven measures of sexual sadism investigated ranged from substantial to almost perfect. Kappa and ICC values of all the 172 variables used can be found in the Electronic Supplementary Materials (Table 8).

We also ascertained the proportion of cases identified as having sexual sadism at different cut-off scores, and used the SeSaS Part 2 as another scale with its score ranging from 0-3. A first step was taken to determine the equivalent cut-off scores on each scale and pragmatically, based on the proportion of sexual homicide cases identified as sexually sadistic in the research literature, we aimed to identify the score yielding a rate of between 1 in 4 or 1 in 3 cases.

Secondly, to ascertain concurrent (i.e., between the same types of measures, e.g., SeSaS Part 1 and SADSEX-SH) and convergent validity (i.e., between different types of measures, e.g., SeSaS Part 1 and RDCSSD), correlations between the seven measures of sexual sadism were calculated.

Thirdly, using all seven measures, rates of sexual sadism in the sample were ascertained. For the SeSaS Part 1, three approaches were taken: the recommended cut-off of 4, the cut-off of 4 after removing Item 1, and the higher cut-off of 5 derived from the two previous steps. For SeSaS Part 2, we used the cut-off of 2 derived from the previous steps. For the MTCSS, the cut-off of 6 derived from the previous steps was used. For RDCSSD, we used three thresholds: definite, probable, and possible.

Fourthly, to ascertain whether the different approaches corresponded with each other beyond being correlated (i.e., whether they identified the same cases), we cross-tabulated the approaches that identified between 1 in 4 and 1 in 3 cases as sexually sadistic and we ascertained percentage agreement and kappa coefficients.

Fifthly and finally, we examined the external validity of sexual sadism as ascertained using the recommended cut-off scores of SeSaS Part 1 and SADSEX-SH, and thus the correlates of sexual sadism in sexual homicide, using bivariate analyses (Chi-square tests and Fisher’s tests). Following Perneger’s (1998) suggestion, using Bonferroni adjustments was deemed unnecessary.

## Results

### Dimensional Scales

#### Descriptives

##### SeSaS

The mean score on Part 1 of the SeSaS was 3.43 (*Mdn* = 3, *SD* = 1.898, range: 0-9). Using the recommended cut-off score of 4, sexual sadism was found in 132 (44.4%) offenders. Table 1 shows that the most prevalent SeSaS items in our sample were expressive physical violence and sexual arousal. The internal consistency of the scale was moderate, with a Cronbach’s α of .62 (DeVellis, 2012).

##### SADSEX-SH

The SADSEX-SH mean score was 4.24 (*Mdn* = 4, *SD* = 2.836, range: 0-14). Sexual sadism as defined by the recommended cut-off of 6 was identified in eighty-eight (29.6%) of cases. As shown in Table 1, the most prevalent SADSEX-SH items were gratuitous/excessive violence and sexual domination. Scale reliability found a Cronbach’s α = .58, indicating moderate internal consistency (DeVellis, 2012).

##### MTCSS

The mean score for the MTCSS was 4.51 (*Mdn* = 4, *SD* = 2.162, range: 0-11). The most prevalent MTCSS items recorded were medical problems requiring a physician and cuts, bruises, and abrasions (see Table 1). A Cronbach’s α = .55 indicated moderate internal consistency (DeVellis, 2012).

**Table 1:** Rates of Endorsement of Individual Items of the Three Dimensional Scales: SeSaS, SADSEX-SH and MTCSS

SeSaS		SADSEX-SH			MTCSS	
Item	*N* (%)	Item	*N* (%)		Item	*N* (%)
	*Present*		*Partially Present*	*Present*		*Present*
1. Sexual arousal during the crime scene actions	172 (69.9)	1. Sexual domination of the victim through the use of bondage, asphyxia, blindfolding, a knife, and so on	48 (18.1)	111 (41.9)	1. Victim tied (control)	70 (25.1)
2. Exertion of power, control, domination	162 (60.7)	2. Physical or psychological torture of the victim	50 (19.0)	44 (16.7)	2. Instrumental aggression: Brutal or damaging beating (aggression)	89 (34.1)
3. Torturing the victim(s)	81 (30.3)	3. The victim forced to verbally or physically engage in sexually degrading, humiliating behaviour	35 (13.6)	41 (15.9)	3. Expressive aggression: Brutal or damaging beating before the sexual assault (aggression)	113 (43.1)
4. Degrading, humiliating behaviour towards the victim	72 (28.8)	4. Gratuitous violence, excessive injury, biting, cutting, or other acts of physical cruelty inflicted on the victim	49 (16.8)	193 (66.3)	4. Expressive aggression: Brutal or damaging beating after the sexual assault (aggression)	90 (36.1)
5. Mutilation of sexual parts of the victim’s body	67 (25.0)	5. Anal or oral sex forced upon the victim	13 (5.9)	36 (16.3)	5. Kicking (aggression)	39 (15.8)
6. Mutilation of other parts of the victim’s body	110 (40.0)	6. Use of an inanimate object(s) to sexually penetrate the victim	5 (2.1)	22 (9.1)	6. Cuts, bruises, and abrasions (aggression)	199 (71.1)
7. Expressive physical violence	240 (83.9)	7. Sexual mutilation of the victim	12 (4.4)	59 (21.8)	7. Burns (aggression)	20 (7.1)
8. Insertion of objects into the victim’s bodily orifices	27 (11.0)	8. Souvenirs or trophies taken from the victim	11 (4.0)	5 (1.8)	8. Medical problems requiring physician (aggression)	285 (96.3)
9. Ritualistic behaviour	21 (7.5)				9. Cruelty to animal (cruelty)	16 (6.8)
10. Confinement of the victim	54 (19.4)				10. Cruelty to people (cruelty)	195 (69.1)
11. Taking trophies	15 (5.4)				11. Sadistic assaults on victim’s genitals/breasts (torture)	78 (29.0)
12. Planned Conduct	137 (49.3)				12. Expressive aggression: Uncontrollable rage and anger leading to mutilation before the sexual assault (torture)	61 (22.9)
13. Indications of sadistic acts in the past beyond the listed offences	66 (29.1)				13. Expressive aggression: Uncontrollable rage and anger leading to mutilation after the sexual assault (torture)	57 (21.5)
14. Arousability to sadistic fantasies or acts (either self-confessed or apparent through witness statements)	82 (38.0)				14. Anal insertion of object (insertion)	17 (6.6)
					15. Vaginal insertion of object (insertion)	18 (7.1)

#### Cut-off Scores for Dimensional Scales

Table 2 shows that using a cut-off of 5 on the SeSaS Part 1, 6 on the SADSEX-SH, 6 on the MTCSS, and 2 or more items on SeSaS Part 2 (although not a dimensional scale as such) led to percentages of around 30%, falling within the expected prevalence rate of sexual sadism in SH cases (1 in 4 to 1 in 3 cases).

Cut-offs for ‘extreme’ scores, identifying the highest scoring 5% of cases, were SeSaS Part 1 ≥ 7, SADSEX-SH ≥ 10, MTCSS ≥ 9; and cut-offs identifying ‘very high’ scores, identifying the highest scoring 15%, were SeSaS Part 1 ≥ 6, SADSEX-SH ≥ 8, MTCSS ≥ 7. About a third of cases below the threshold for sexual sadism (SeSaS Part 1 3-4, SADSEX-SH 3-5, MTCSS 4-5) could be considered ‘sub-threshold’; while the bottom third (SeSaS Part 1 0-2, SADSEX-SH 0-2, MTCSS 0-3) could be considered ‘non-sadistic’.

**Table 2:** Prevalence of Sexual Sadism at Different Cut-off Scores on Dimensional Scales With Indicative Percentiles

SeSaS Part 1		SeSaS Part 2		SADSEX-SH		MTCSS		Indicative percentile
Score	% identified	Score	% identified	Score	% identified	Score	% identified	
0	100.0	0	100.0	0	100.0	0	100.0	0.0
						1	98.5	2.0
1	94.9			1	92.1	2	93.4	10.0
2	85.9			2	83.9	3	82.9	20.0
								30.0
3	64.7			3	67.2	4	63.9	35.0
		1	59.2	4	59.0			40.0
						5	48.9	50.0
4	44.4							55.0
				5	38.2			60.0
5	28.7	2	28.9	6	30.0	6	30.5	70.0
				7	20.4	7	18.6	80.0
6	16.6			8	14.6			85.0
		3	11.4			8	10.4	90.0
				9	7.1			92.5
7	4.8			10	5.4	9	5.0	95.0
8	2.2			11	2.3			97.5
				12	1.6			98.0
9	0.7			13	0.6	10	0.6	99.0
				14	0.3	11	0.3	99.5
10-11	0.0			15-16	0.0	12 -15	0.0	100.0

*Note.* The lines indicate the cut-off scores for a rate of sexual sadism close to 30% of cases, within the expected range of 1 in 4 to 1 in 3.

### Different Measures of Sexual Sadism

#### Convergent and Concurrent Validity

Correlations between the seven measures of sexual sadism are reported in Table 3. Almost all of the measures were correlated with each other with at least a medium effect size, with the correlation of SeSaS Part 1 and SADSEX-SH indicating a huge effect. The only two measures that were poorly and not significantly correlated were MTCSS and clinical diagnosis.

**Table 3:** Correlation Matrix for Seven Measures of Sexual Sadism

Sexual sadism measures	1	2	3	4	5	6	7	8
1. CDSSD	–	.487***	.341***	.334***	.138*	.142*	.096	.292***
2. RDCSSD		–	.503***	.428***	.425***	.376***	.234***	.596***
3. MTC:R3/ CM3ST			–	.586***	.304***	.340***	.193***	.481***
4. SMT				–	.347***	.336*	.124*	.480***
5. SeSaS Pt.1					–	.781***	.554***	.356***
6. SADSEX-SH						–	.535***	.279***
7. MCTSS							–	.189**
8. SeSaS Pt2								–

*Note.* CDSSD = clinical diagnosis of sexual sadism disorder; RDCSSD = research diagnosis of coercive sexual sadism disorder; MTC:R3/CM3ST = sadistic type of rapist or child molester (Knight et al., 1989; Knight 1989); SMT = sexualised murder type (Higgs et al., 2017).

*p < .05. **p < .01. ***p < .001.

Scatterplots (see Figure 1, 2, and 3 in the Electronic Supplementary Materials) show huge and very large effect sizes in the correlations between the three dimensional scales. In keeping with the results in Table 2, they confirmed that a SeSaS Part 1 score of 5, a SADSEX-SH score of 6, and a MTCSS score of 6 appear to be equivalent.

#### Rates of Sexual Sadism

Table 4 shows the rates of sexual sadism identified by the seven measures of sexual sadism. We included different approaches for SeSaS (cut-off of 4 and 5, and exclusion of Item 1) and RDCSSD (using the higher threshold level of probable/definite, and lower threshold level ranging from possible to definite diagnosis). The expected range was 1 in 4 to 1 in 3 cases from the research literature.

**Table 4:** Prevalence Rates of Sexual Sadism According to Different Measures

Measure	Sadism criterion	*n/N*	*%*
Clinical diagnosis	Clinical diagnosis of SSD by psychiatrist or psychologist (CDSSD)	11/297	3.7
Research diagnosis	Research diagnosis of probable/definite CSSD (RDCSSD)	54/297	18.2
	Research diagnosis of possible/probable/definite CSSD (RDCSSD)	93/297	31.3
MTC typology	Rapist (MTC:R3) or child molester (MTC:CM3) sadistic type (MTC:R3/CM3ST)	54/297	18.2
Higgs typology	Sexualised murder type (SMT)	79/297	26.6
SeSaS Part 1	SeSaS Part 1 score of 4 or more	132/297	44.4
	SeSaS Part 1 score of 5 or more	85/297	28.6
	SeSaS Part 1 (without item 1) score of 4 or more	104/297	35.0
SeSaS Part 2	SeSaS Part 2 score of 2 or more	87/297	29.3
SADSEX-SH	SADSEX-SH score of 6 or more	88/297	29.6
MTCSS	MCTSS score of 6 or more	89/297	30.5

In our sample, using the Higgs SMT led to a rate that fell at the lower end of the expected range. Seven approaches led to rates of about 1 in 3: possible RDCSSD, SeSaS Part 1 cut-off of 5, SeSaS Part 1 cut-ff of 4 after removing item 1, SeSaS Part 2 cut-off of 2, SADSEX-SH cut-off of 6, and a MTCSS cut-off of 6. A higher than expected rate, of almost 1 in 2, was found using the recommended SeSaS Part 1 cut-off of 4.

#### Correspondence Between the Measures

The next step was ascertaining whether these measures were equivalent and corresponded with each other, namely if they identified the same cases. Measures which identified rates of sexual sadism within the expected range were therefore cross tabulated with each other, and percentage agreements and kappa coefficients were calculated (see Table 5). The highest agreement was found for the SeSaS Part 1 cut-off of 5 and the SADSEX-SH cut-off of 6, with almost 9 in 10 agreement and a substantial kappa value, indicating that the two scales at these cut-offs are indeed identifying the same people. The SeSaS Part 1 cut-off of 5 showed the best agreement and highest Kappas with other measures overall.

**Table 5:** Correspondence Between Different Criteria for Sexual Sadism

First Criterion	Second Criterion	Sadist on Both	Sadist on First Only	Sadist on Second Only	Non-sadist on Both	Agreement %	Kappa
SeSaS 5	SADSEX-SH 6	69 (23.5)	15 (5.1)	19 (6.5)	190 (64.8)	88.3	0.72
SeSaS 5	MTCSS 6	48 (16.5)	36 (12.2)	41 (13.9)	169 (57.5)	74.0	0.37
SeSaS 5	RDCSSD	52 (17.5)	33 (11.1)	41 (13.8)	171 (57.6)	75.1	0.41
SeSaS 5	SMT	39 (13.1)	46 (15.5)	40 (13.5)	172 (57.9)	71.0	0.28
SADSEX-SH 6	MTCSS 6	50 (17.1)	38 (13.0)	39 (13.4)	165 (56.5)	73.6	0.38
SADSEX-SH 6	RDCSSD	27 (9.2)	61 (20.8)	27 (9.2)	178 (60.8)	70.0	0.19
SADSEX-SH 6	SMT	39 (13.3)	49 (16.7)	38 (13.0)	167 (57.0)	70.3	0.27
MTCSS 6	RDCSSD	27 (9.2)	62 (21.1)	26 (8.8)	179 (60.9)	70.1	0.20
MTCSS 6	SMT	31 (10.5)	58 (19.7)	46 (15.6)	159 (54.1)	64.6	0.13
RDCSSD	SMT	33 (11.1)	21 (7.1)	46 (15.5)	197 (66.3)	77.4	0.36

*Note.* SeSaS 5 = SeSaS Part 1 score of 5 or more; SADSEX-SH 6 = SADSEX-SH score of 6 or more; MTCSS 6 = MTCSS score of 6 or more; RDCSSD = possible research diagnosis of coercive sexual sadism disorder; SMT = Higgs et al. (2017) sexualised murder type.

### External Validity of SeSaS and SADSEX-SH Sadism

Table 6 (see Electronic Supplementary Materials document) shows the bivariate analyses comparing sadistic and non-sadistic SHOs regarding offender characteristics, using the recommended cut-offs for the SeSaS Part 1 of 4 and the SADSEX-SH of 6 to define offenders as sadistic. SeSaS sadism was correlated with the presence of personality disorders (in particular, psychopathy) and paraphilic disorders (in particular, coercive sexual sadism disorder), and not having mental impairments. SADSEX-SH sadism was only correlated with lack of mental impairment, psychopathy, and coercive sexual sadism disorder.

Table 7 (see Electronic Supplementary Materials document) shows the same bivariate analyses but for offence-related variables. SeSaS sadism was correlated with the degree of planning of the offence, post-mortem sexual activity, strangulation or stabbing as causes of death, using or involving a weapon, unusual acts, crime scene control, taking precautions to avoid detection, choosing a stranger victim, having a co-offender, being a serial killer, and long offences. Using the SADSEX-SH led to the identification of similar offence-related correlates: the degree of planning, ejaculation, post-mortem sexual activity, using or involving weapon, taking precautions to avoid detection, and having a co-offender.

## Discussion

### Main Findings

We investigated the assessment of sexual sadism in a large sample of SHOs from Australia and New Zealand using data gathered primarily from legal documents. Our main aim was to provide practitioners with practically relevant information to guide them in assessing and diagnosing sexual sadism in SHOs. Given the questionable reliability of clinical diagnosis of sexual sadism and recommendations to use dimensional behavioural scales, we investigated the application of these scales, how they compared to and were related to each other, as well as diagnostic and typological approaches. In the Electronic Supplementary Materials, two tables comparing our findings using the SeSaS with those of other studies (Tables 9 and 10) are included. Our replication of very similar findings regarding the offender and offence characteristics associated with sexual sadism (ascertained with the SeSaS and SADSEX-SH) as in studies from Europe and North America provides confirmation of these correlates and indicates generalisability of our findings.

All but one of the correlations between the seven measures were significant, with the dimensional scales showing concurrent validity with each other and convergent validity with typological and diagnostic approaches. Our findings confirm problems with relying on clinical diagnosis (Marshall & Kennedy, 2003; Marshall et al., 2002; Nitschke et al., 2013), and indicate more structured approaches, particularly typological ascertainment and behavioural scales, were superior. The former may be due to typologies providing structured guidance and a limited number of possibilities akin to making a differential diagnosis, and the latter due to scales being dimensional and providing objective behavioural criteria.

From previous findings, we expected a rate of sexual sadism between 1 in 4 and 1 in 3. The recommended SeSaS Part 1 cut-off of 4 led to a rate well above the expected range, suggesting three possibilities. Firstly, the rate and degree of sexual sadism may be higher in our sample. Secondly, the SeSaS may have been over scored in the current study. Thirdly, the cut-off score of 4 may be too low in sexual homicide cases. Darjee (2019) and Stefanska et al. (2019) previously found lower rates of 26.9% and 29.4% using this cut-off, while Chopin et al. (2022) found a similar rate of 45.5%. It was previously suggested that a cut-off of 4 might be too low (Gonçalves et al., 2020; Higgs et al., 2019; Mokros et al., 2012; Stefanska et al., 2019), particularly in sexual homicide cases where items such as sexual arousal (Item 1), exertion of power, control domination (Item 2), and excessive physical violence (Item 7) are very common (Darjee, 2019; Stefanska et al., 2019). Although these items may describe manifestations of sadism, they may also be present where sadism is absent (Proulx et al., 2021). Indeed, in SH samples (the current one; Darjee, 2019; Stefanska et al., 2019) these three items are as or more common than in samples only made up of sexual sadists (e.g., Chopin et al., 2022).

Using a possible threshold for a diagnosis of ICD-11 CSSD, the Higgs sexualised murderer type, a SADSEX-SH cut-off of 6, a cut-off of 6 on the MTCSS, increasing the SeSaS cut-off to 5, and keeping the recommended SeSaS cut-off of 4 while removing Item 1 led to rates in line with the expected range. The very high correlation between SeSaS Part 1 and the SADSEX-SH, their identification of corresponding cases (with cut-offs of 5 and 6 respectively), and their very similar correlates, indicates they are equivalent measures. This is unsurprising given that seven of the eight items of the SADSEX-SH are identical or almost identical to items of SeSaS Part 1.

We found relatively high rates of the three SeSaS Part 2 items in our sample. Unfortunately, other studies do not examine these items either individually or in combination. Having two of the three Part 2 items identified in the middle of the expected range. Part 2 correlated with all the other measures, and interestingly showed the highest correlations with research diagnosis and typological approaches, perhaps confirming its more clinical focus. Items 13 and 14 seem very specific clinical indicators of sexual sadism: the presence of one of these appears highly indicative of sexual sadism, and the absence of these may question the presence of sexual sadism, especially in cases with borderline scores on Part 1.

### Limitations

The present study is not without limitations. The main limitation is the data sources. Although we collected data from reliable sources (i.e., official Court reports), each case document was different in terms of the amount, quality, and clarity of the information reported, with older cases usually reporting less information than more recent ones. Consequently, some variables could not be coded in every case due to a lack of sufficient information, leading to missing data in the dataset. This problem with the data sources may be partly responsible for the low rates of clinically diagnosed sexual sadism disorder and lack of information to ascertain research diagnoses of coercive sexual sadism disorder.

In addition, all cases were coded by postgraduate psychology and psychiatry students. The accuracy and reliability of the coding, especially concerning the assessment of mental disorders and sexual sadism, may reflect the lack of professional experience compared to an experienced clinician or researcher. To counter this, researchers were trained, regularly discussed cases with an experienced senior researcher, and inter-rater reliability was checked for all cases. Although we achieved generally good levels of reliability across the measures of sexual sadism, they were lower than those reported from some other studies.

### Implications

The present study supports the use of dimensional behavioural scales in the assessment and ascertainment of sexual sadism in SHOs. Given the number of studies on the SeSaS Part 1, this should be the preferred scale. The equivalence of the SADSEX-SH, however, would tend to indicate that the two can be used interchangeably. Although we suggest an MTCSS score of 6 for identifying sexual sadism in sexual homicide offenders, there is a dearth of empirical studies to support its clinical use at present.

We suggest that practitioners use the following approach in sexual homicide cases. Firstly, apply SeSaS Part 1, using a score of 5 as highly indicative of sexual sadism. A score of 4 is potentially indicative, 3 indicates sexual sadism is unlikely, and 6 is very highly indicative. In general, a score of 2 rules out the diagnosis while 7 rules it in. The practitioner should then use Part 2 and a typological approach, particularly if the Part 1 score is between 3 and 6, to determine whether the individual is sexually sadistic. Finally, having taken these steps, consideration can be given to whether the individual is diagnosed with DSM-5 sexual sadism disorder or ICD-11 coercive sexual sadism disorder. Taking these structured steps should bring reliability, validity, and utility to the diagnosis of sexual sadism, with a combination of an assessment and diagnostic approach which follows a structured clinical judgment process.

A question that should be addressed in future research is whether the cut-off score for sexual sadism in sexual homicide is different, and higher, than that in non-homicidal sexual offending. It may be that the scores on sadism scales are equivalent in homicidal and non-homicidal cases, but due to the number of individuals who score relatively highly for other reasons (e.g., grievance, anger, multiple perpetrators), the primary issue is differentiating people who score highly due to sadism from people who score highly for other reasons. Differentiating such cases may involve identifying factors which are highly specific to sexual sadism but unusual in non-sadistic sexual violence (e.g., ritualism, object insertion, sexually sadistic arousal or fantasy). Alternatively, it may be that due to the common baseline behaviour in sexual homicide (e.g., brutal aggression, sexual behaviour, domination), scores are automatically elevated in both sadists and non-sadists. Another possibility is that sexual sadism is different in severity or in its nature in those who kill compared to those who commit non-fatal offences (Chopin et al., 2022). All these potential scenarios have implications for how latent sexual sadism is assessed with dimensional scales that examine overt crime scene behaviours.

## Supplementary Materials

The Supplementary Materials include the following items (for access, see Consigliere et al., 2025S):

- Scatter plots showing the correlations of the dimensional behavioural scales (Figures 1, 2 and 3)
- Comparison of sadistic and non-sadistic offenders (as determined by the developers’ recommended SeSaS and SADSEX-SH cut-offs) on relevant offender and offence variables (Tables 6 and 7)
- Reliability coefficients for all relevant study variables (Table 8)
- SeSaS findings from current study compared with previous studies (Tables 9 and 10)

ConsigliereL.
KneißlerL. Z. M.
NanevA.
DavisM.
DarjeeR.
 (2025S). Supplementary materials to "Assessing sexual sadism in sexual homicide offenders using behavioural dimensional scales, typological approaches, and categorical diagnoses"
[Additional figures and tables]. PsychOpen. 10.23668/psycharchives.21219
PMC1291459841716191

## Data Availability

Data is available from authors on request.

## Author Note

The data set (or parts of it) used for the current study have been used in other published studies which have examined different research questions. None of these have specifically investigated the assessment of sexual sadism in our sample and so do not address the same research questions. The aforementioned manuscripts are: Ricono-Kaufhold et al. (2024) on child sexual homicide, Czarnietzki et al. (2024) on intimate partner sexual homicide, de Oliveira et al. (in press) comparing sexual homicide to non-sexual homicide, Clarkson et al. (2020) on multiple perpetrator sexual homicide, and Eichinger and Darjee (2021) an initial descriptive study. Studies are also in progress with this sample examining serial sexual homicide, stranger sexual homicide, home invasion sexual homicide, mutilation in sexual homicide, and validating and developing typologies.
